# Assessment of Pharmacokinetics and Toxicology of Sadat-Habdan Mesenchymal Stimulating Peptide (SHMSP) in Rats and Goats

**Published:** 2014-09

**Authors:** Ibrahim Al-Habdan, Mir Sadat-Ali, Mastour Safar Alghamdy, Akram Randhawa, Shahanas Chathoth

**Affiliations:** 1 Department of Orthopaedic Surgery, University of Dammam, Saudi Arabia;; 2 Department of Pharmacology, University of Dammam, Saudi Arabia;; 3 Prince Mohammed bin Fahd Centre for Research and Consultation Studies, University of Dammam, Saudi Arabia

**Keywords:** Sadat-Habdan Mesenchymal stimulating peptide, Toxicology, Pharmacokinetics

## Abstract

**Background:**

Sadat-Habdan Mesenchymal Stimulating Peptide (SHMSP) was discovered and patented with USPTO in 2008. Studies have shown that SHMSP works as an angiogenesis factor. This study was done to evaluate pharmacokinetics (PK) in rats and toxicology studies in goats and rats.

**Methods:**

In 80 skeletally mature Sprague Dawley rats 5 milligram/kg body weight of SHMSP was injected intramuscularly. Blood samples were collected at 0, 30, 60, 120, 240, 320 minutes and 480 minutes. The plasma calibration curves were prepared at concentrations of 6.25, 3.12, 1.56, 0.78 and 0.39 ng/mL by spiking 190 µL of rat plasma with 10µL of working standard and 200 µL of deionized water. Samples were vortexed for five seconds, centrifuged at 14000 rpm for 30 minutes at 4°C and the supernatant was collected analyzed using High-performance liquid chromatography (HPLC). After injection of 20 µL sample, the peptide was eluted with 15ml linear gradient up to 36% of eluent A. The time between injections was 25 min. and the eluent was monitored at a wavelength of 215 nm. The concentration of peptide present in the rat plasma samples collected at different time intervals were quantified using standard curve method. The goats were injected deep intramuscularly 100 mg/kg-body weight of the SHMSP dissolved in injection solution. In 10 Sprague Dawley rats of ≥250 grams of weight, 20 mg/kg/day SHMSP was injected for 7 consecutive days. All the animals were kept at a close watch. Clinical observation at least once daily and as necessary was undertaken. After 2 weeks animals were euthanized and major organs were harvested and histopathology samples were obtained and processed.

**Results:**

There were no deaths is either of the study and control group of animals. The gross observations of the various organs appeared normal and histopathological studies did not show any toxicity in the organs tested.  The plasma concentration-time profile of SHMSP after intramuscular injection, the level of SHMSP in an initial high phase reaching the highest at 30 minutes 2.3184 ng/ml and 60 minutes 1.7447 ng/ml at 60 minutes. The lowest level was at 360 minutes of 0.0879 ng/ml.

**Conclusions:**

The dose of SHMSP at 20 times the recommended dose was not toxic and secondly the peak time and level was at 30 minutes to 120 minutes and the plasma half-life of SHMSP was 90 minutes.

## INTRODUCTION

Biological factors were discovered to enhance healing of tissues including bone and became a subject of intense ongoing investigations. Many growth factors have been isolated which are claimed to enhance wound healing. Growth factors which are undergoing clinical trials include PDGF, FGF-2, IGF, KGF and PDGF is currently approved for use in humans (1). Growth factors such as Platelet-rich plasma (PRP), Bone morphogenic proteins (BMPs), Vasculo endothelial growth factor (VEGF) and Transforming growth factor (TGF-β) have been found to have effect on fracture healing (2-6). Bone morphogenic proteins were reported to be factors that can induce transformation of mesenchymal cells into chondroblasts and osteoblast (7, 8).

SHMSP was discovered and patented in 2008 as a 13-amino acid peptide. Initial studies were focused on fracture healing. The preclinical studies for SHMSP was carried out for simple fracture healing and in iatrogenic critical size defect of the forearm bone of rabbits which showed excellent healing power of SHMSP (9, 10). The initial assessment was that SHMSP was found to be act like an angiogenesis factor (11). Recently Al-Hoqail *et al* (12) found the SHMSP showed better healing of the wounds in burn sites and Al-Elq *et al* (13) in the iatrogenic wounds in diabetic rabbits.

This study was done to assess the preclinical safety in non-rodent species (goat) and rodents and pharmacokinetics in a rodent species before initiating Phase I human clinical trials as a new drug in patients with delayed fracture healing and to enhance would healing.

## METHODS

Ninety (90) Sprague Dawley adult male rats and twenty adult male goats were acquired and identified by markings and were acclimated to the study environment for 16-21 days prior to dose administration. Animals were individually housed in suspended wire caging, and were kept on a 12 h / 12 h light/dark cycle except when interrupted for study procedures. Average room temperature was regulated in the range 18 to 29°C, average relative humidity of 30-70%, and an average daily airflow >10 fresh air changes/h. Animals were fed Lab Diet Certified Rodent Diet 5002 Meal food ad libitum, except during fasting prior to dose administration, and had access to water ad libitum.

In 90 skeletally mature male Sprague Dawley rats 5 milligram/kg body weight SHMSP was injected intramuscularly. Blood samples from the treated rats were collected after different time interval such as 30, 60, 120, 180, 240, 360, 480 and 1440 minutes. Samples from non-injected group were collected as control samples. Blood was transferred into heparinized tubes, the protease inhibitor then immediately added. The samples were stored at −80°C until analysis.

The goats were injected deep intramuscularly 100 mg/kg body weight of the SHMSP dissolved in injection solution. In 10 Sprague Dawley rats of over 250 grams 20 mg/kg/day for 7 consecutive days was injected. All the animals were kept at a close watch. Clinical observation at least once daily and as necessary was undertaken. After 2 weeks animals were euthanized and major organs were harvested and histopathology samples were obtained and sent for histopathological studies.

### Standards and Sample preparation

Primary stock solution of standard was prepared by dissolving lyophilized standard compound in deionized water to get a concentration of 1000 ng/mL and stored at -20°C. Working standard solutions were prepared by serially diluting the stock solution in deionized water to obtain the linear concentrations 250, 125, 62.5, 31.25 and 15.625 ng/mL. The plasma calibration curves were prepared at concentrations of 6.25, 3.12, 1.56, 0.78 and 0.39 ng/mL by spiking 190 µL of rat plasma with 10 µL of working standard and 200 µL of deionized water at an appropriate concentration (to make up to 400 µL of total sample volume). All these procedures were conducted on ice. Samples were vortexed for five seconds, centrifuged at 14000 rpm for 30 minutes at 4°C and the supernatant was collected for the analysis. Rat plasma samples were collected from the treated rats at different time points and prepared the samples for analysis.

### HPLC Analysis

**Figure 1:**
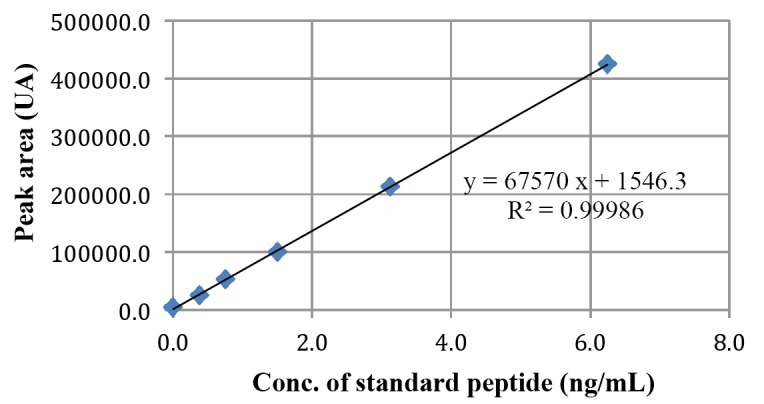
Standard curve obtained using serially diluted known concentration of the standard peptide.

Acetonitrile (HPLC grade, Fischer Scientific) and Milli-Q water was used for the analysis. Analysis was performed using HPLC Waters 2535 quaternary gradient module system with Waters 2489 UV detector and Waters 2707 auto-sampler. Separations were carried out on a Waters BioSuite C18 analytical column (4.6 × 250 mm) connected to a guard (3.9 × 20 mm) column and the system was operated at an ambient temperature. The column was equilibrated with 76% eluent A (water) and 24% eluent B (acetonitrile) with both containing 0.1% trifluoroacetic acid at a flow rate of 1.0 mL/min as mobile phase. After injection of 20 µL sample, the peptide was eluted with 15ml linear gradient up to 36% of eluent A. The time between injections was 25 min. and the eluent was monitored at a wavelength of 215 nm. Plasma was isolated, processed and 20 μL was injected to HPLC system. The concentrations obtained after three animals injections have been tabulated as Run 1, Run 2 and Run 3 (Table 1). The concentration of peptide present in the rat plasma samples collected at different time intervals were quantified using standard curve method. The unknown concentrations of peptide present in the plasma samples were calculated applying the value of peak area in the equation obtained from the standard curve (Figure 1). Data shown are mean ± SD (n=3).

Ten goats were injected deep intramuscularly 100 mg/kg body weight of the SHMSP dissolved in injection solution. In 10 Sprague Dawley rats of over 250 grams 20 mg/kg/day for 7 consecutive days was injected. All the animals were kept at a close watch. Clinical observation at least once daily and as necessary was undertaken. After 2 weeks animals were euthanized and liver, kidneys, spleen, brain, heart and intestines were harvested and sent for histopathological studies.

## RESULTS

**Figure 2:**
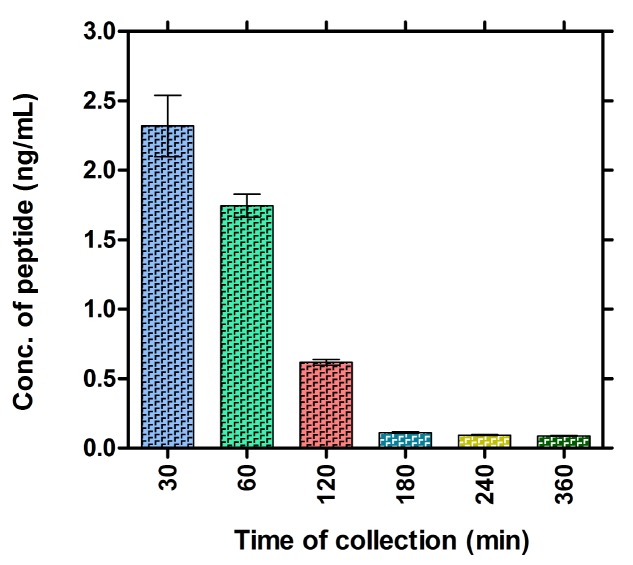
Concentration of the plasma level of SHMSP between 30-360 minutes.

There were no deaths is either of the study group or in control animals. Blood samples from the treated rats were collected after different time interval at 0, 30, 60, 120, 180, 240, 360, 480 and 1440 minutes (24 hours). Samples from non-injected group were collected as control samples. Plasma was isolated, processed and 20 uL was injected to HPLC system. The peak areas obtained for the three different animals has been tabulated (Table 1). The peptide concentration of samples collected at each time points were calculated using the peak area “designated as “y” and solving the equation on (y = 67570 “x” + 1546.3) obtained from the standard curve (Figure 1). The concentration of peptide present in the plasma collected at different time points was quantified and plotted by keeping time on X-axis and the concentrations on Y-axis (Figure 2). Data shown are mean ± SD. The maximum plasma concentration (C_max_) can be seen in sample collected at 30 minutes after IM injection and the concentration of peptide was 2.32 ng/mL. A gradual decline of concentration such as 1.74, 0.62 and 0.11 ng/mL was seen in the following samples collected at 60, 120 and 180 minutes later to injection, respectively (Figure 2). This plot indicates the peptide required less than half an hour to reach to the blood stream and then it is degraded gradually. After 180 minutes, the concentration of peptide was almost equivalent to that of the control indicating complete degradation of the peptide. In our experiment the initial concentration (at 30 min) of the peptide was 2.31839 ng/mL. The control data was not shown in the figure as we did not observe any peak area at the expected retention time.

**Table 1:** The concentrations obtained for three different rats at each time points and their average value

S. No.	Time points (min)	Run 1 (ng/mL)	Run 2 (ng/mL)	Run 3 (ng/mL)	Average conc. of peptide (ng/mL)

1	30	2.2753	2.7208	1.9590	2.3184
2	60	1.7819	1.8659	1.5862	1.7447
3	120	0.6140	0.6527	0.5841	0.6169
4	180	0.1154	0.1022	0.1165	0.1114
5	240	0.0930	0.0899	0.0987	0.0939
6	360	0.0936	0.0814	0.0898	0.0879

**Figure 3:**
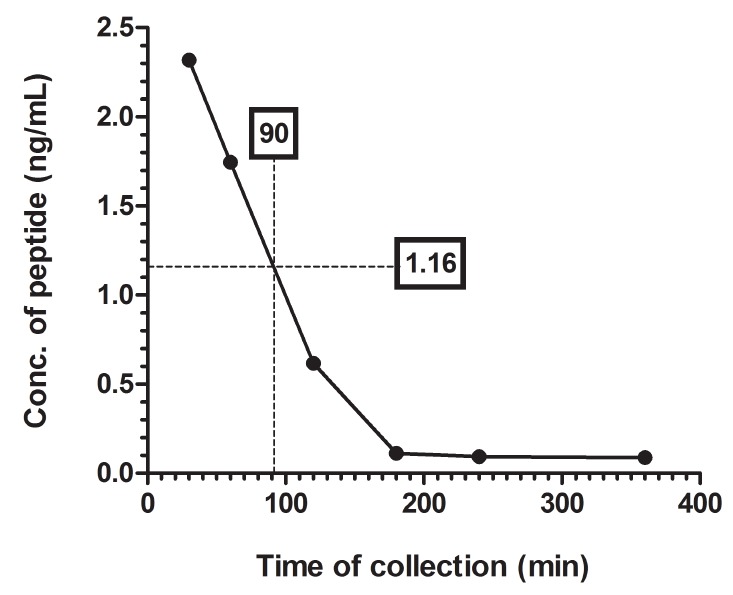
Plasma Half-Life of SHMSP

The peptide reached to the half of its initial concentration (i.e. 1.16 ng/mL) at 90 min making the plasma half-life of the peptide from the plot is 90 min (Figure 3).

**Figure 4:**
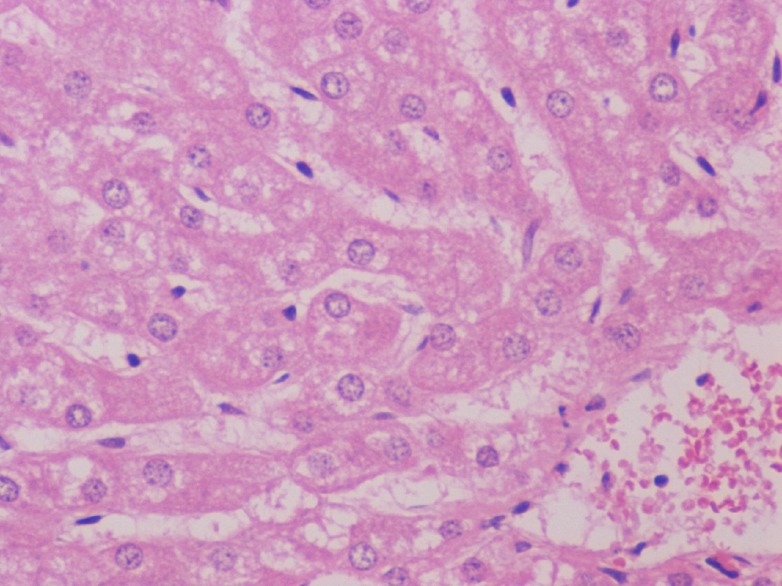
Liver with no histologic abnormalities. H & E × 400.

**Figure 5:**
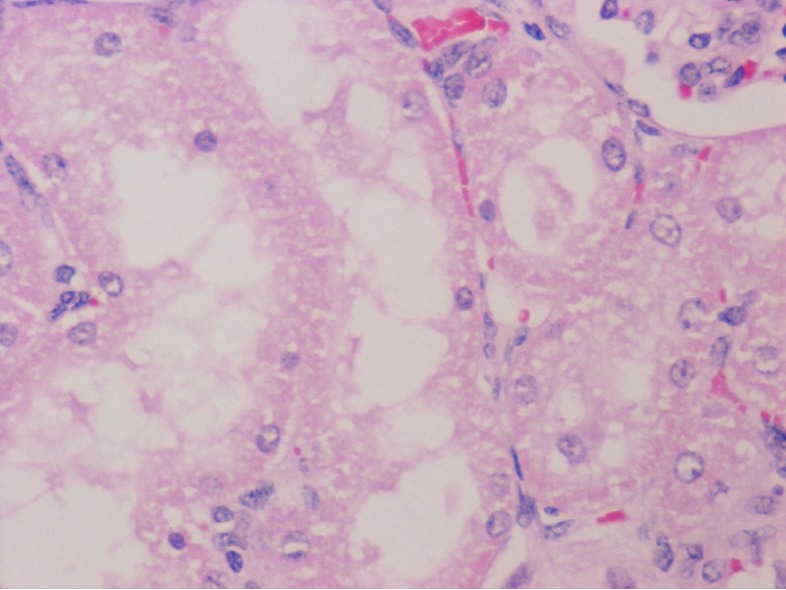
H & E × 400 of the kidney and Renal tubules showing no abnormal histological changes.

**Figure 6:**
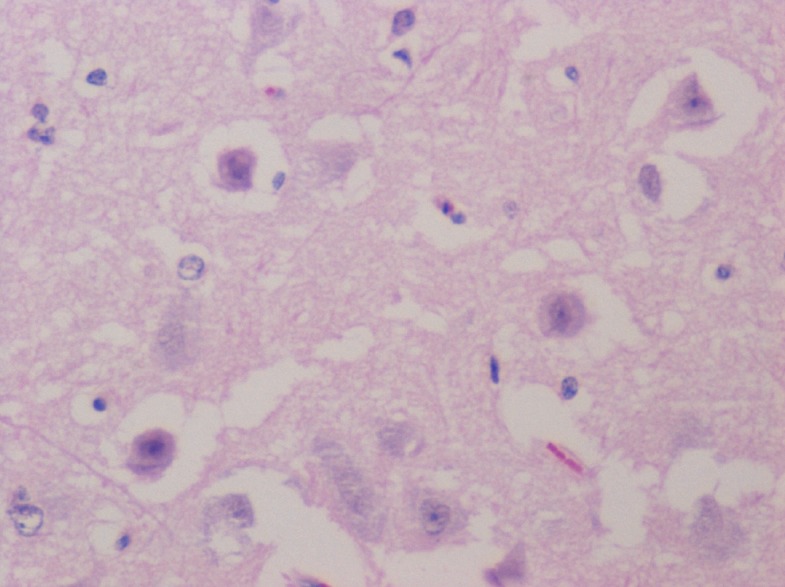
H & E × 400 section of the brain tissue which does reveal any abnormalities due to the SHMSP peptide injection.

The gross observations of the various organs in the rodent and non-rodent animals appeared normal and histopathological studies did not show any toxicity in the organs tested (Figures 4, 5, 6).

## DISCUSSION

This study showed that SHMSP at the recommended dosage level of 5 mg/kg body weight reached a peaked in 30 minutes and remained in the circulation for 180 minutes and secondly at a 20 times the therapeutic dose did not cause any toxic effects on the major organs of the tested animals. Since only one route bolus intramuscularly was chosen for both the animals (Rats and goats) administered and found to be well tolerated without onset of systemic toxicity. The route of administration of SHMSP tested till now in preclinical studies was direct instillation/application at the site and a bolus single dose was appropriate to test the levels in the serum and toxicology studies. The safety of SHMSP and in commination of collagen in animal studies demonstrated in a comprehensive way that there was no evidence of any adverse effects when the peptide was used.

Angiogenesis is important in the growth, development and recovery from the disease process, contrary to this anti-angiogenesis has received more attention due to control of cancer growth. Vasculo endothelial growth factor (VEGF) an angiogenic growth factor was studied to heal chronic wounds and decreased vascularity. VEGF, a glycoprotein induces angiogenesis, helps in increasing glomerular capillary hyper-permeabilty and underlies the pathogenesis of diabetic nephropathy (14, 15). Topical VEGF was shown to accelerates diabetic wound healing by way of enhanced angiogenesis (16), and as the SHMSP stimulates angiogenesis the action is similar to VEGH.

The plasma level of SHMSP was maintaining a high level of 1.8 ng/ml at 60 minutes, but a carrier like collagen can increase duration of the exposure of the peptide to the targeted tissues and expand the use for tissue engineering applications. Three of the pro- angiogenic growth factors up for clinical trials have a short half-life. The half-life of VEGF was reported to be under 90 minutes in the circulation (17). Recently Kleinheinz *et al* (18) showed that when VEGF165 was mixed with a collagen complex charged with the 80 µg of VEGF165 showed measurable cytokine release even after 48 hours. A carrier complex need to be developed for SHMSP for delayed release and longer bioavailability if required.

In conclusion, this study evaluated for any potential toxicity after a single administration of SHMSP in goats and rats and found none. The biological activity in rats showed after the single injection of 5 mg/kg body the peak was between 30 and 60 minutes and total excretion by 180 minutes.
